# A Reliability of Electromyographic Normalization Methods for the Infraspinatus Muscle in Healthy Subjects

**DOI:** 10.2478/hukin-2013-0007

**Published:** 2013-03-28

**Authors:** Sung-min Ha, Heon-seock Cynn, Oh-yun Kwon, Kyue-nam Park, Gyoung-mo Kim

**Affiliations:** 1 Department of Physical Therapy, Division of Health Science, Baekseok University, Cheon-an, Republic of Korea.; 2 Applied Kinesiology and Ergonomic Technology Laboratory, Department of Physical Therapy, College of Health Science, Yonsei University, Won-ju, Republic of Korea.; 3 Laboratory of Kinetic Ergocise Based on Movement Analysis, Department of Physical Therapy, College of Health Science, Yonsei University, Won-ju, Republic of Korea.

**Keywords:** electromyography, infraspinatus muscle, normalization, reliability

## Abstract

The purpose of this study was to examine the test-retest reliability of normalization methods for the infraspinatus muscle in a group of healthy subjects. Twelve healthy subjects (male=8, female=4) performed the maximal voluntary isometric contraction (MVIC) with examiner`s resistance, MVIC with a digital tension-meter (MVIC-DT), and sub-MVIC methods. Surface electromyography (EMG) signals were recorded from the infraspinatus muscles according to normalization methods. Reliability was analyzed using the intra-class coefficient (ICC), standard error of measurement (SEM), and minimal detectable difference (MDD). The results of the present study demonstrated that the sub-MVIC method has excellent test-retest reliability (ICC=0.92) with a relatively small SEM (5.9 mV) and MDD_95_ (16.4 mV), compared to MVIC-DT (ICC=0.73; SEM=11.2 mV; MDD_95_: 31 mV) and MVIC-E (ICC=0.5; SEM=15.7 mV; MDD_95_: 43.6 mV). These findings provide evidence that sub-MVIC is more appropriate for comparing the EMG activity for the infraspinatus muscle as a normalization method. If MVIC for normalization is needed, MVIC-DT is more appropriate than MVIC-E.

## Introduction

The infraspinatus muscle produces an approximation force to resist distraction during an overhead throwing motion (Ballantyne et al., 1993). Also, the infraspinatus provides the primary external rotation force (Terry and Chopp, 2000). Because of its critical role in providing dynamic stability and producing external rotation torque at shoulder joint, many authors have advocated emphasis on infraspinatus muscle strengthening during rehabilitation or athletic conditioning programs in order to enhance muscular strength and endurance (Blackburn et al., 1990; Brewster and Schwab, 1993; Reinold et al., 2004; Townsend et al., 1991).

Previous studies were conducted using surface electromyography (EMG) to measure the muscle activity of infraspinatus through EMG studies in a variety of exercises (Ballantyne et al., 1993; Reinold et al., 2004). A major limitation of kinesiologic EMG research is the difficulty in making comparisons between EMG values obtained from identical muscles in different subjects, different muscles from the same subject, or even the same muscle from the same subject on different days. These difficulties may be due to subtle differences in muscle architecture, electrode placement, and electrode construction (Giroux and Lamontagne, 1990; Jonsson and Komi, 1973; Kadaba et al., 1985). To overcome these shortcomings of surface EMG, the concept of normalization has been developed to enable comparing EMG signal (Mirka, 1991).

Numerous studies have been performed using maximal voluntary isometric contraction (MVIC) normalization method to identify effect of exercises or intervention for infraspinatus muscle strengthening (Ballantyne et al., 1993; Bitter et al., 2007; Ekstrom et al., 2003). The MVIC normalization technique is the use of the maximal voluntary contraction of a predetermined isometric movement as the reference EMG signal (Hagberg and Sundelin, 1986; Yang and Winter, 1983). The MVIC has the advantage of having a physiological meaning where derived data are expressed relative to the maximum (Allison et al., 1998). To produce MVIC, resistances of examiner’s hand or digital tension-meter (DT) have been used as a common method (Kendall and McCreary, 2005; Netto and Burnett, 2006). As the reference value for normalization, MVIC may account for much of the potential variability among recording factors (e.g. skin impedance, electrode position, collection methods and devices, electrode size and pick-up area, etc.). However, the reproducibility of this reference point depends on subject`s level of sincerity, motivation, or pain during the exertion. The subjective nature of these exertions may introduce some level of experimental error (Marras and Davis, 2001). Larivière et al. (2002) demonstrated that between-day reliability of MVIC method was poor in the comparison of EMG activities for the back muscles between healthy control subjects and chronic patients with low back pain.

To address these limitations, sub-MVIC is frequently used as a predetermined reference value when MVIC are limited by aging, pain or other symptoms (Allison et al., 1998; Dankaerts et al., 2004; Marras and Davis, 2001). This approach is limited by the difficulty of establishing equivalent sub-maximal loads for different muscles (Allison et al., 1998). To establish equivalent sub-maximal loads, the estimation of the expected maximum contraction, 60% MVIC using isokinetic dynamometer was used to predict a reference point to be used for normalization in the neck and trunk muscles (Burnett et al., 2007; Dankaerts et al., 2004; Marras and Davis, 2001; Netto and Burnett, 2006). Previous studies reported that sub-MVIC are more reliable and are more sensitive than MVIC when assessing low levels of abdominal muscle activities (Allison et al., 1998; O’Sullivan et al., 1998). Also, Sub-MVIC has been reported to be reliable within-day reliability in healthy subjects when assessing EMG for abdominal wall muscles (Allison et al., 1998; O’Sullivan et al., 1998). However, there is no attempt to investigate sub-MVIC for the infraspinatus muscle, compared to MVIC.

Therefore, the purpose of this study was to examine the reliability of normalization methods for the infraspinatus muscle in a group of healthy subjects. Specifically, MVIC with examiner`s resistance (MVIC-E) as a common method, MVIC with digital tension-meter (MVIC-DT), and sub-MVIC methods were examined. The hypothesis of this study was that sub-MVIC method would more reliable than other normalization method.

## Material and Methods

Twelve healthy subjects (male=8, female=4) were recruited from the university populations. The characteristics of the subjects are presented in Table 1. There were significant differences in physical characteristics between males and females. Inclusion criteria were 1) ability to perform full shoulder external rotation comfortably, 2) manual muscle testing (MMT) grade was 5/5 (Hislop and Montgomery, 2002; Kendall and McCreary, 2005). Exclusion criteria were past or present neurological, musculoskeletal, or cardiopulmonary diseases that could interfere with shoulder external rotation in the testing position. Before the study, the principal investigator explained all procedures to the subjects in detail. All subjects signed an informed consent form, which was approved by the Yonsei University Wonju Campus Human Studies Commities.

EMG data were collected using a Noraxon TeleMyo 2400T and analyzed using MyoResearch Master Edition 1.06 XP software (Noraxon Inc., Scottsdale, AZ, USA). Skin preparation of electrode sites involved shaving and cleaning with rubbing alcohol. Surface electrode pairs were positioned at an interelectrode distance of 2 cm. The reference electrode was placed on the ipsilateral clavicle. EMG data were collected for the infraspinatus muscle (4 cm below the spine of the scapula, on the lateral aspect over the infrascapular fossa of the scapula) (Cram et al., 1998). The raw signal was full wave rectified and filtered using a Lancosh FIR digital filter. The bandpass filter was used between 20 Hz and 300 Hz. The EMG data were processed into the root mean square (RMS) value, which was calculated from 50-ms windows of data points.

**Figure 1:**
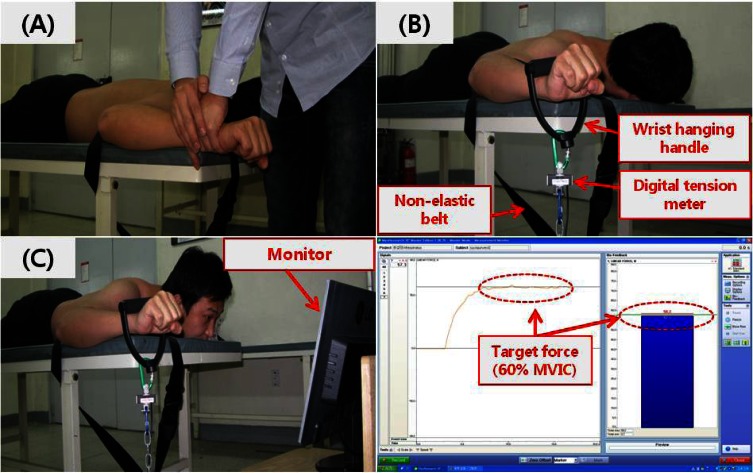
Testing Position: A: MVIC-E (maximal voluntary isometric contraction with examiner’s resistance); B: MVIC-DT (maximal voluntary isometric contraction with digital tension-meter); C: Sub-MVIC (sub-maximal voluntary isometric contraction)).

The digital tension-meter using linear force measurement load cell (Noraxon Inc., Scottsdale, AZ, USA) was used to measure maximal force and 60%-maximal force (kg) in the infraspinatus muscle. The force data were collected using a Noraxon TeleMyo 2400T and MyoResearch Master Edition 1.06 XP software (Noraxon Inc., Scottsdale, AZ, USA). The target force to calculate 60%-MVIC (sub-MVIC) was determined based on the maximal force value using digital tension-meter. The sampling rate was 1000 Hz. The digital tension-meter was calibrated prior to each set of measurement (Figure 1).

### Procedures

The dominant arm (the tendency to prefer a particular arm in performing selected tasks) was tested in all subjects (Yoshizaki et al., 2009). Recent findings have suggested that the determination of a dominant arm was based on hand-path kinematics and muscle activity in performing selected tasks (Bagesteiro and Sainburg, 2002). However, our study used a questionnaire to self-report arm dominance (ex. Daily-use dominant arm). Their self-reported dominant arms were right in all subjects.

Testing position required the subject to lay prone with the shoulder abducted at 90° and the elbow flexed to 90°, while the forearm in neutral position (Kendall and McCreary, 2005). Then, the subject moved to a position of shoulder external rotation to 90°. In a pilot study, isometric external rotation at 0° and 90° shoulder abduction were chosen because these positions are known to generate high levels of activity in the infraspinatus muscle (Kronberg et al., 1990; Jenp et al., 1996; Reinold et al., 2004). Standing or sitting external rotation at 0° and 90° shoulder abduction had a compensatory trunk motion compared to the prone position. Therefore, the prone position with the shoulder abducted at 90° was chosen. Additionally, the subject`s elbow was fastened to the table using a non-elastic belt to prevent compensatory shoulder motion (Figure 1). Subjects were familiarized with each normalization trials during the 30 min period prior to testing. The familiarization period was completed when the subject was able to maintain three normalization methods for 5 s. All of the subjects were comfortable after the familiarization period, and none reported fatigue. A 15 min rest period was allowed after familiarization period before data collection began. The order of testing was randomized using random number generator (Microsoft Corp., Redmond, WA, USA), except for sub-MVIC. Sub-MVIC was calculated after MVIC-DT trial.

Three different testing methods were examined in this study (Figure 1). For the MVIC-E trial, subjects performed the maximal contraction of the dominant side arm by applying a manual resistance of an examiner to the subject`s wrist. All subjects were given consistent verbal encouragement during maximal contraction. For the MVIC-DT trial, each subject performed a maximal contraction of the dominant side arm using a wrist hanging handle. The handle was connected to the digital tension-meter. After MVIC-DT trial, a sub-MVIC value was calculated to 60% MVIC-DT force (Netto and Burnett, 2006). For the sub-MVIC trials, subjects were provided with visual feedback from a computer monitor that was positioned directly in the subject’s line of sight to assist them in achieving the desired level of contraction. Each trial being performed incorrectly was stopped and repeated. If the subject performed the test incorrectly over 5 times, he or she was asked to rest 1 hour to prevent learning effects.

Physiological recovery was facilitated by allowing a 2 min recovery between normalization trials (Burnett et al., 2007). EMG activity was measured during each normalization trials for 5 s. The first and last second of the EMG data from each trial were discarded, and the remaining 3 s of data were used for further analysis (Reinold et al., 2004). An hour after the first session, the subject performed the second session following the identical protocol.

### Statistical analysis

A repeated measure ANOVA was used to determine if there was systemic bias (for confirming the learning effects) between the first and second trial. Reliability of normalization methods in the infraspinatus muscle was calculated to determine the within-subject variation using two indices of reliability; ICC (3,1), the standard error of measurement (SEM). ICC values were calculated using the Statistical Package for Social Sciences, Version 12.0 (SPSS Inc, Chicago, IL). ICC is commonly used to assess test–retest reliability and reflects the relative reliability of a measurement. ICC >0.75 is considered excellent, 0.40–0.75 is regarded as fair to good, and 0–0.4 as poor (Crossley et al., 2004). To examine the consistency of the measurement, the SEM was calculated using Microsoft Excel [SEM = standard deviation*(1-ICC)^1/2^]. Minimal detectable difference (95% confidence interval) (MDD_95_) scores were calculated [MDD_95_ = SEM × √2 × 1.96] (Ries et al., 2009). MDD_95_ scores using a Microsoft Excel (set at a 5 % significance level) were calculated for the three normalization methods.

## Results

There were no significant differences (*p* >0.05) between the first and second test session in all of the normalization methods. These results indicated that the learning effect did not occur between test sessions. The same day test–retest ICC scores, SEM, and MDD_95_ for the EMG recordings from the infraspinatus muscle during each normalization test are documented in Table 2. The maximal and sub-MVIC force data using digital tension meter are presented in Table 3.

## Discussion

The purpose of this study was to determine optimal normalization methods for the infraspinatus muscle in healthy subjects. The results of present study demonstrated that the sub-MVIC method has excellent test-retest reliability (ICC = 0.98) with a relatively small SEM (1.3 mV) and MDD_95_ (3.5 mV), compared to MVIC-DT (ICC = 0.73; SEM = 6.3 mV; MDD_95_: 17.3 mV) and MVIC-E (ICC = 0.42; SEM = 14.5 mV; MDD_95_: 40.3 mV). Consistent with results of the present study, it has been previously reported that sub-MVIC methods were more reliable than MVIC in healthy controls when examining EMG data from biceps femoris and triceps muscles (Allison et al., 1993; Yang and Winter, 1983).

Several possible explanations exist for our results. First, providing visual bio-feedback at the reference point (60% MVIC torque) may have reduced the variability of measurement in the sub-MVIC method, compared with no visual feedback (MVIC-DT and MVIC-E). Previous studies suggested that providing visual feedback through monitor at the reference point markedly increased the reliability of the normalization method in the sub-MVIC (60% MVIC) (Burnett et al., 2007; Netto and Burnett, 2006). Second, the differences between MVIC-DT and MVIC-E are influenced by methods of applying resistance. Although the same investigator applied manual resistance during the MVIC-E, the use of manual resistance is a potential source for variability (Dankerts et al., 2004). In contrast to MVIC-E, MVIC-DT is applied by fixed wrist hanging handle. During the measurement of MVIC, this method is useful for reducing variability introduced by the manual resistance method. Thus, MVIC-DT has higher reliability with a relatively small SEM and MDD_95_ than MVIC-E.

Although MVIC is the most commonly used normalization technique, the MVIC may vary depending upon the sincerity, motivation or pain level of the individual. This variability may result in substantial MVIC variability and influence the interpretation of the EMG signal (Marras and Davis, 2001). Also, it is limited in application because it applies only to healthy subjects and requires substantial rest periods and thus, significant time. MVIC techniques would also have limited utility when evaluating individuals who are suffering from pain since they may not be willing to generate “true” MVICs (Baratta et al., 1998). Lund et al. (1991) reported that pain reduces maximal muscle activation, but has no influence on sub-maximal muscle activation in patients with musculoskeletal pain. Therefore, we suggest that sub-MVIC is appropriate for normalization during EMG studies, compared to MVIC. If MVIC for normalization is needed, MVIC-DT is more optimal than MVIC-E.

The present study had some limitations. First, our results are not widely generalizable because all of our subjects were the healthy males. Thus, additional research is needed to establish whether our findings apply to subjects with shoulder pain as well as female subjects. Second, we did not measure between-day reliability. Between-days reliability becomes critical when assessing EMG parameters that are used as outcome measures (Elfving et al., 1999). However, it has been suggested that replacing the electrodes may be a major source of between-days test–retest variance, even if these are intended to be identically re-positioned (Veiersted, 1991). In conclusions, the present study demonstrated that sub-MVIC method using a providing visual bio-feedback at the reference point (60% MVIC torque) has excellent test-retest reliability in the infraspinatus muscle, compared to MVIC methods. This study also demonstrated that MVIC-DT is more reliable than MVIC-E. These findings provide evidence that sub-MVIC is more appropriate for comparing the EMG activity for the infraspinatus muscle as a normalization method. If MVIC for normalization is needed, MVIC-DT is more appropriate than MVIC-E.

## Figures and Tables

**Table 1:** Characteristics of the subjects (N=12)

	**Total**	**Male (n=8)**	**Female (n=4)**	***p***
**Body height (cm)**	170.7 ± 7.3	174.9 ± 4.5	162.3 ± 2.1	0.00
**Body mass (kg)**	68.5 ± 15.5	76.6 ± 12.2	52.3 ± 1.7	0.03
**Age (years)**	26.0 ± 4.5	29.0 ± 3.1	21.3 ± 0.5	0.01

**Table 2:** Test-retest ICC scores, SEM, and MDD_95_ among three methods.

	MVIC-E	MVIC-DT	Sub-MVIC
ICC	0.42	0.73	0.98
SEM	14.5 mV	6.3 mV	1.3 mV
MDD_95_	40.3 mV	17.3 mV	3.5 mV

ICC = intraclass correlation coefficient; MDD = minimal detectable difference; SEM = standard error of measurement; MVIC-E = maximal voluntary isometric contraction with examiner`s resistance; MVIC-DT = maximal voluntary isometric contraction with digital tension-meter; Sub-MVIC = sub-maximal voluntary isometric contraction.

**Table 3:** The force data using a digital tension meter.

	MVIC-DT		Sub-MVIC	

	1^st^ trial	2^nd^ trial	1^st^ trial	2^nd^ trial
Force (kg)	56±14.9	54.1±15.4	32.4±8.2	32.3±8.5

MVIC-DT = maximal voluntary isometric contraction with digital tension-meter; Sub-MVIC = sub-maximal voluntary isometric contraction.
